# Draft Genome Assembly of *Floccularia luteovirens*, an Edible and Symbiotic Mushroom on Qinghai-Tibet Plateau

**DOI:** 10.1534/g3.120.401037

**Published:** 2020-02-25

**Authors:** Xiaolong Gan, Dong Cao, Zhenyu Zhang, Shu Cheng, Le Wei, Shiming Li, Baolong Liu

**Affiliations:** * Key Laboratory of Adaptation and Evolution of Plateau Biota, Northwest Institute of Plateau Biology, Chinese Academy of Sciences, Xining, Qinghai, 810008, China; † University of Chinese Academy of Sciences, Beijing, 100049, China; ‡ Key Laboratory of Crop Molecular Breeding, Xining, Qinghai, 810008, China; § BGI-Shenzhen, Shenzhen, 518083, China; ** College of Biologic and Geographic Sciences, Qinghai Normal University, Xining, Qinghai, 810008, China

**Keywords:** *Floccularia luteovirens*, Genome assembly, CAZymes, Phylogenetic analysis, Species-specific genes, Genome report

## Abstract

*Floccularia luteovirens*, also known as “Yellow mushroom”, is an edible ectomycorrhizal fungus widely distributed in the Qinghai-Tibet Plateau alpine meadow. So far, little genomic information is known about *F. luteovirens*, which is not conductive to the protection and utilization of it. In this manuscript, we present a first draft genome assembly and annotation of *F. luteovirens*. The fruiting body of *F. luteovirens* was sequenced with PacBio Sequel and Illumina Hiseq 2500 system. The assembled genome size was 28.8 Mb, and comprising 183 contigs with a N50 contig size of 571 kb. A total of 8,333 protein-coding genes were predicted and 7,999 genes were further assigned to different public protein databases. Besides, 400 CAZymes were identified in *F. luteovirens*. Phylogenetic analysis suggested that *F. luteovirens* should belong to the Agaricaceae family. Time tree result showed that the speciation of *F. luteovirens* happened approximately 170 Million years ago. Furthermore, 357 species-specific gene families were annotated against KEGG and GO database. This genome assembly and annotation should be an essential genomic foundation for understanding the phylogenetic, metabolic and symbiotic traits of *F. luteovirens*.

*Floccularia luteovirens*, previously interpreted as *Armillaria luteovirens* or *Tricholoma luteovirens*, an ectomycorrhizal fungus that produces edible and medicinal products, is widely distributed in the alpine meadow in Qinghai-Tibet Plateau (QTP) where the altitude ranges from 3200 to 4800 m. Because the color of its fruiting body is majorly yellow, it also was known as “Yellow Mushroom”. The yellow mushroom is delicious, and rich in abundant nutrients including fibers, amino acids, vitamins and many other nutrients (Jiao *et al.* 2008). It is an important food and financial source for local Tibetans, and considered as one of the “Eight Treasures” in Qilian countryside, Qinghai province, China (Xing *et al.* 2018). However, like the other famous fungus in QTP, *Ophiocordyceps sinensis*, *F. luteovirens* still can’t be cultivated artificially. It usually forms a symbiont with *Kobresia humilis*, playing crucial roles in the QTP alpine meadow ecosystem. In the fruiting season, its fruiting body will generally form a fairy ring with a stimulation zone. The plants growing on the fairy ring are flourishing and dense, and the vegetation is dark green. It is probably because the abundant bacteria and mycelium under the fairy ring is conductive to the growth of *K. humilis* (Xing *et al.* 2018). Although the previous researchers had carried out the analysis of the phylogeographic, microbiology, and genetic diversity of *F. luteovirens* (Xing *et al.* 2017; Xing *et al.* 2018), the taxonomic status of *F. luteovirens* is still uncertain, and the molecular mechanism of special traits is also unclear.

The high-speed development of advanced sequencing techniques has facilitated the exploration of the mechanisms of fungal growth, metabolism, and evolution at molecular level. At present, massive genomic information of fungus have been provided at the Ensembl and Genebank database including fungus with high medicinal value like *Ganoderma lucidum* (Chen *et al.* 2012; Liu *et al.* 2012) and *Daedalea quercina* (Nagy *et al.* 2016), edible fungus like *Volvariella volvacea* (Bao *et al.* 2013), *Flammulina velutipes* (Kurata *et al.* 2016), *Agaricus bisporus *(Morin *et al.* 2012; Sonnenberg *et al.* 2016), and so on. With the help of the available genome sequences, researchers could not only ascertain evolutionary relationship of different fungus (Bushley *et al.* 2013; Xia *et al.* 2017), but also preliminarily elucidate the molecular mechanism of special traits such as: color, flavor, and so on. However, the genome sequences of *F. luteovirens* is still lacking. In order to effectively investigate the genetic characteristics of it, the genome was assembled and annotated for the first time in this study.

## Materials And Methods

### Fungus collection and DNA extraction

The fruiting bodies of *F. luteovirens* were collected in alpine meadow at Qilian countryside, Qinghai province, China. The specimen used for the genome sequencing was deposited in the key laboratory of molecular breeding in Northwest Institute of Plateau Biology (NWIPB), Chinese Academy of Sciences (CAS), with accession number NWIPB-YM1807. Genomic DNA of the fruiting body was extracted from 10 μg specimen using the CTAB method (Watanabe *et al.* 2010). The DNA concentration was determined using NanoDrop 2000 spectrophotometer (Thermo Fisher Scientific, USA), and the integrity of the DNA was detected using 1.0% agarose gel.

For RNA sequencing (RNA-seq), RNA of the fungal cap and stipe (consistent with the specimen in DNA sampling) was extracted with TRIzol Reagent (Invitrogen, Carlsbad, CA, USA). After removing residual DNA and rRNA, reverse transcription was carried out and the pooled cDNA libraries were sequenced using the Illumina HiSeq 2500 platform with 150 bp paired-end reads. All the samples were sequenced with three biological replicates.

### Genome sequencing and assembly

The genome of *F. luteovirens* was sequenced by high-throughput PacBio Sequel platform and Illumina HiSeq 2500 system (Genewiz Biotechnology Co. Ltd., Suzhou, China). The PacBio long reads could provide a better sequencing length, meanwhile the Illumina paired-end reads have lower mismatch rate. Thus, the hybrid sequencing approach was used for ensuring a high coverage and integrity of genome. PacBio long reads with 2.5 kb library were generated by PacBio Sequel platform. For paired-end reads, 150 bp insert library was constructed with Illumina HiSeq 2500 system. To make sure the accuracy of follow-up analysis, we filtered the adapters, low-quality reads, and N reads from raw data using SOAPnuke v1.5.6 (-l 10 -q 0.2 -n 0.001) (Chen *et al.* 2018). First, the genome size and hybrid rate of *F. luteovirens* was estimated through kmerfreq (https://github.com/fanagislab/kmerfreq) analysis using Illumina reads. Then, the filtered PacBio subreads (>2 kb) were used for initial assembly which was performed using Canu software (Koren *et al.* 2017) with the parameters: genomeSize = 30m minReadLength = 2000 minOverlapLength = 1000 useGrid = 0 corOvlMemory = 15 for several times. After comparison, we selected a better assembly with longer N50. The selected assembly was further polished using BWA v0.7.17 (Li and Durbin 2009) in mem algorithm mode and GATK v3.3.0 (parameters:–genotyping_mode DISCOVERY -stand_emit_conf 10 -stand_call_conf 30) (https://software.broadinstitute.org/gatk/) with Illumina reads. At last, the completeness of the final genome assembly was assessed using Benchmarking Universal Single-Copy Orthologs program v3.0 (Simão *et al.* 2015) based on basidiomycota_odb9 database. In addition, the genome assembly of *F. luteovirens* was compared with another two fungi in Agaricaceae and three fungi in Tricholomataceae.

### Genome annotation

Before the gene model prediction, repeat sequences of the assembled *F. luteovirens* genome were identified in two ways including *de novo* method and homology-based annotation. In *de novo* method, RepeatModeler v1.0.11 (http://www.repeatmasker.org/RepeatModeler/), LTR FINDER (Xu and Wang 2007) and RepeatMasker v4.0.6 (Tarailo-Graovac and Chen 2009) were adopted to find the repeat sequences. In homology-based annotation, RepeatMasker and RepaetProteinMasker v4.0.6 (Tarailo-Graovac and Chen 2009) were used to search the repats against the Repbase database (Bao *et al.* 2015). Furthermore, Tandem Repeat Finder v4.07b (Benson 1999) was employed to dectect the tandem repeats. For non-coding RNAs, tRNAscan-SE v1.23 (Gardner *et al.* 2009), RNAmmer v1.2 (Lagesen *et al.* 2007), and Rfam v9.1 (Boeckmann *et al.* 2003) were used to identify the tRNA, rRNA, and snRNA, respectively.

Gene models in the *F. luteovirens* genome were identified using sequence homology method, *de novo* gene prediction method and along with the RNA-seq data. For the sequence homology method, protein sequences of related species: *Agaricus bisporus*, *Armillaria ostoyae*, *Armillaria gallica*, *Lepista nuda*, *Macrolepiota fuliginosa*, *Tricholoma matsutake* were downloaded from the NCBI and JGI databases. The protein sequences were aligned to our genome assembly with tblastn (E value cut-off: 1e-05). Different protein BLAST hits were merged. Then, the complete gene structure was predicted with GeneWise (Birney *et al.* 2004) according to the corresponding gene region. For the *de novo* gene prediction method, three *de novo* prediction programs: AUGUSTUS v3.3.1(Keller *et al.* 2011), geneid v1.4.4 (Blanco *et al.* 2007), and glimmerhmm v3.0.1 (Majoros *et al.* 2004) were employed to train and predict gene models. Furthermore, RNA-seq data of each sample were aligned to our genome assembly using HISAT2 v2.0.4 (Kim *et al.* 2015). Then, the spliced reads were assembled into transcripts with Cufflinks v2.2.1 (http://cole-trapnell-lab.github.io/cufflinks/install/) to define the protein-coding gene models. Eventually, all the resulting evidences were integrated to a comprehensive and non-redundant reference gene set using EVidenceModeler v1.1.1 (Haas *et al.* 2008) with a weight distribution of RNA-seq(6), homology(5), *de novo*(3).

Functional annotation of the predicted genes was actualized with various protein databases: InterProScan (Jones *et al.* 2014), Eukaryotic Orthologous Groups (KOG) (Tatusov *et al.* 2003), Non-redundant Protein Database (Nr) (Yu and Zhang 2013), Kyoto Encyclopedia of Genes and Genomes (KEGG) (Kanehisa *et al.* 2017), Gene Ontology (GO) (Ashburner *et al.* 2000), TrEMBL (O’Donovan *et al.* 2002) and Swiss-Prot (Magrane 2011). Annotation of Carbohydrate-Active Enzymes (CAZYmes) in the *F. luteovirens* genome was identified using hmmscan (HMMER 3.1b2 http://hmmer.org/) with E value cut-off of 1e-10 on the basis of dbCAN database (http://csbl.bmb.uga.edu/dbCAN/).

### Gene family classification and phylogenetic analysis

To further understand the evolutionary relationship of *F. luteovirens*, coding sequence (CDS) file of 30 species distributed in 14 families were downloaded from NCBI and JGI for phylogenetic analysis. We used OrthoMCL v1.4 (Chen *et al.* 2006) to identify the matched protein pairs and cluster gene families. The CDS were first translated into protein sequences. Then, the all-*vs.*-all BLASTP was performed with E-value cut-off of 1e-05. Similar protein matches were obtained with match length cut-off was 80%. The potential ortholog pairs in different species, in-paralog pairs, co-ortholog pairs were identified with E-value cut-off of 1e-05. And the pairs file was further clustered into different gene families using MCL program with parameters:–abc -I (inflation number) 1.5. For phylogenetic analysis, all the single-copy gene families were extracted by a Perl script. First, multiple alignments of protein sequences were conducted using MUSCLE-3.8.31 (Edgar 2004) with default parameters; then, a CDS alignment was actualized based on the former protein alignments. Subsequently, all aligned CDSs were concatenated to generate a supergene for each species using a Perl script. At last, we extracted the nucleotides at position 2 (phase 1) of each codon to construct the phylogenetic tree using RAxML v8.2.11 (Stamatakis 2014) with GTRGAMMA mode. After fast and slow Maximum likelihood (ML) tree searching, one optimized ML tree were sequentially constructed with the bootstrap value of 100. And *Stereum hirsutum* was chosen as the outgroup. Besides, in order to investigate the speciation and divergence time of these fungi, we used the Langley-Fitch (LF) approach (divtime method = lf algorithm = tn) in r8s v1.70 (Sanderson 2002) to construct the time tree based on the final ML tree and one calibration. The output tree file was corrected artificially, and the final time tree was generated by MCMCtree program in PAML v4.9 (Yang 2007). Evolutionary timescale of *Armillaria gallica-Armillaria ostoyae* (Median Time: 12.0 Mya), *Agaricus bisporus-Macrolepiota fuliginosa* (Median Time: 64 Mya), *Pleurotus ostreatus-Stereum hirsutum* (Median Time: 224 Mya) were obtained from Timetree public knowledge-base (Kumar *et al.* 2017). In addition, gene families in *Tricholoma matsutake*, *Macrolepiota fuliginosa*, *Agaricus bisporus*, and *Floccularia luteovirens* were further collected for comparative analysis. The result of it was summarized in Venn diagram using the Perl scripts and the species-specific genes of *F*. *luteovirens* were further screened out and annotated with KEGG and GO databases.

### Data availability

The whole genome sequence of *F. luteovirens* described here has been deposited at GenBank under the accession number RPFY00000000.1. The NCBI BioProject and BioSample ID are PRJNA505547 and SAMN10425398, respectively. The RNA-seq data were also uploaded in NCBI with BioProject of PRJNA602038 and BioSample of SAMN13883324. All the supplemental materials have been uploaded in GSA Figshare. Figure S1 shows the quality distribution of Illumina paired-end reads. Figure S2 shows the Length distribution of the PacBio long reads. Figure S3 shows four K-mer analysis result of *Floccularia luteovirens* genome. Figure S4 shows BUSCO assessment result of *F. luteovirens* genome assembly. Figure S5 shows the distribution of orthologous genes across 30 Basidiomycetes species. Figure S6 shows the time tree of 30 Basidiomycetes species. Figure S7 shows the Venn diagram of gene family distribution between four related fungi. Table S1 shows the sequencing data statistics of *F. luteovirens*. Table S2 shows the K-mer analysis of *F. luteovirens* genome. Table S3 shows the statistics of transposon elements in *F. luteovirens*. Table S4 shows the brief genome comparison between *F. luteovirens* and other fungi. Table S5 shows the functional annotation of predicted genes. Table S6 shows the comparative analysis of Carbohydrate-active enzymes between *F. luteovirens* and *A. bisporus*. Table S7 shows the statistics of 30 Basidiomycetes species in phylogenetic tree. Table S8 shows the statistics of orthologous genes across 30 Basidiomycetes species. Supplemental material available at figshare: https://doi.org/10.25387/g3.11494371.

## Results And Discussion

### Genome assembly

In total, 171.93 × long reads (5.20 Gb) and 169.69 × paired-end reads (5.27 Gb) were generated using the PacBio Sequel and the Illumina HiSeq 2500 sequencing platform, respectively (Table S1). After filtration and correction, the trimmed paired-end reads were divided into quarters for K-mer frequency distribution analysis. Four 17-mer results consistently suggested that *F. luteovirens* genome was ∼30.7 Mb, with a slightly hybrid (Table S2, Figure S3). Table 1 illustrated genome features and gene models of the genome assembly. The final assembled genome size was 28.7 Mb with G+C content reached 43.36%. By comparing 1335 searched BUSCOs, there were 1254 (93.9%) completed BUSCOs and 38 (2.9%) fragmented BUSCOs in *F. luteovirens*, which showed a fairly good completeness of our genome assembly (Figure S4).

**Table 1:** Gene annotation statistics of *Floccularia luteovirens*

Genome features & Gene models	Value
Total length (bp)	28,778,388
Contigs (#)	183
N50 (kb)	571
G+C content (%)	43.36
Gene models (#)	8,333
Average gene length (bp)	1,982
Average length of coding sequence (bp)	1,526
Average exon per gene (#)	6.87
Average exon length (bp)	222
Average intron length (bp)	66.39

### Genome annotation

Repeat sequences prediction showed that the total length of repeats was 3.7 Mb, covering 12.93% of the genome. Transposon elements (TEs) make up a large part of the total (12.14%). These TEs include retrotransposons (Class I) and DNA transposons (Class II). Among the class I TEs, the proportion of long interspersed nuclear elements (LINE), long interspersed nuclear elements (SINE), and long terminal repeats (LTRs) accounted for 0.22%, 0.01%, and 9.47% of the genome, respectively. In addition, the DNA transposons occupied 1.12% and other unknown type were about 1.75% of the assembled genome (Table S3). The predominant transposon type of *F. luteorivens* is LTRs which are considered as the engine of gene and genome evolution (Galindo-González *et al.* 2017). In addition, 149 tRNAs, 72 rRNAs and 10 snRNAs were identified in all, accounting for 1.2% of the genome. This means that the ncRNAs are a very small percentage of the *F. luteorivens* genome.

Combing RNA sequencing data, homology and *de novo* prediction methods, a total of 8,333 protein-coding genes were predicted in the *F. luteorivens* genome. The length of genes accounted for 57.4% (16,519,509) of the genome sequence, with an average length of genes being 1,982 bp. Besides, the average length of exons and introns was 222 bp and 66.39 bp, respectively (Table 1), which is similar to *Agaricus bisporus* (Morin *et al.* 2012). We also collected brief genome information of another two fungi in Agaricaceae and three fungi in Tricholomataceae for comparison. It was worth to note that both the genome size and the number of predicted genes of *F. luteovirens* were smaller than others (Table S4). For example, there were 11,289, 15,801, 14,880, 22,885 and 23,810 predicted genes in the genome of *Agaricus bisporus*, *Macrolepiota fuliginosa*, *Lepista nuda*, *Tricholoma matsutake*, and *Tricholoma terreum*, respectively, while only 8,333 genes could be detected in *F. luteovirens*. In addition, the fungal genome size in Agaricaceae is quite smaller than in Tricholomataceae on average.

Functional annotation of the predicted genes was carried out against various public protein databases such as the InterPro, SwissProt, KEGG, GO and so on. Of the 8,333 predicted genes, 7,999 (95.99%) genes could be assigned to the databases mentioned above. More specifically, 5,954 (71.45%), 5,156 (61.87%), 5,435 (65.22%), 4,138 (49.66%), 4,680 (56.16%), 7,993 (95.92%), and 3,834 (46.01%) genes were annotated with InterPro, Nr, Swiss-Prot, KEGG, KOG, TrEMBL, and GO database, respectively (Table S5).

### CAZymes analysis

**Figure 1:**
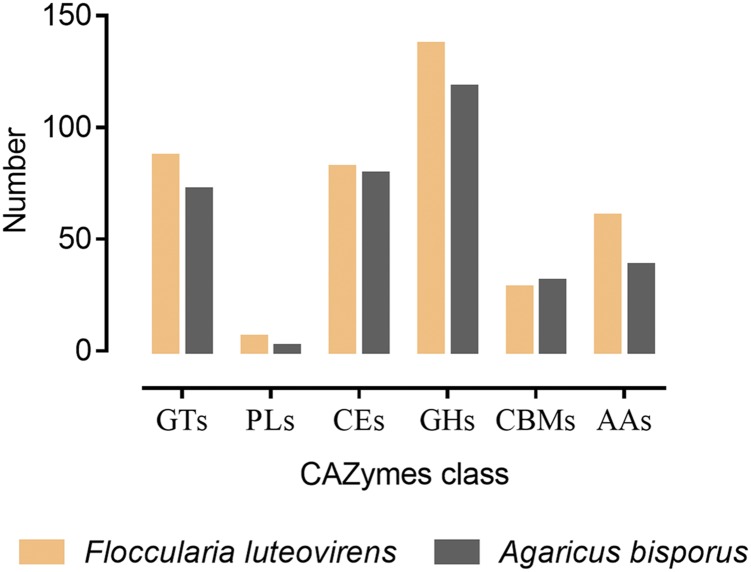
Comparative analysis of CAZymes between *Floccularia luteovirens* and *Agaricus bisporus*.

To investigate the genes involved in degradation of organic matter in *F. luteovirens*, the genome was further annotated with dbCAN database using HMMER program. CAZymes paly a big role in modification, and degradation of cellulose, hemicellulose, and lignin (Morin *et al.* 2012; Sánchez 2009), consisting of 6 classes: Glycoside Hydrolases (GHs), Glycosyl Transferases (GTs), Polysaccharide Lyases (PLs), Carbohydrate Esterases (CEs), Auxiliary Activities (AAs), and Carbohydrate-Binding Modules (CBMs). Total 400 genes were assigned to CAZyme families, accounting for 8.58% of the genome. The GHs (137) were the most predominant CAZymes, implying that polysaccharides and cellulose decomposition were crucial for symbiotic growth of *F. luteovirens* in QTP alpine meadow (Table S6). Because GHs are responsible for catalyzing the hydrolysis of the glycosidic linkage of glycosides. We further compared the CAYmes of *A. bisporus*, a widely cultivated and saprophytic fungus, with *F. luteovirens*. In general, The CAZymes in *F. luteovirens* were more than *A. bisporus* except CMBs (Figure 1, Table S6). These quantity and composition difference of CAZymes between them should contribute to their specific lifestyle and characteristics enabling them adapt to its biotope.

### Gene family classification and phylogenetic analysis

**Figure 2:**
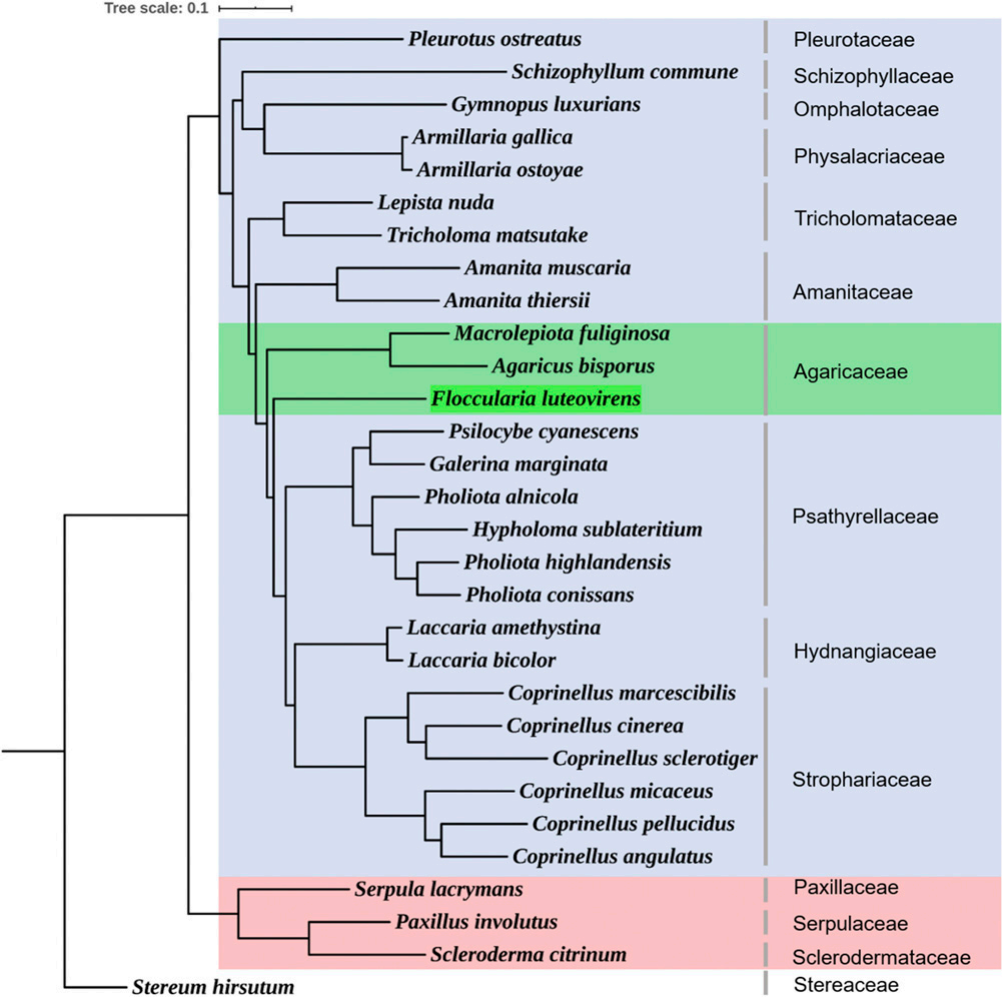
Phylogenetic tree. The fungus in Agaricales and other orders were colored in blue and pink, respectively. Among the Agaricales, the Agaricaceae fungi was colored in green.

In order to investigate the evolutionary relationship of *F. luteovirens*, the genome set of 30 different fungi were collected for constructing the phylogenetic tree (Table S7). We identified orthologous genes and clustered gene families across the 30 fungi. The distribution of identified orthologs, paralogs, and unclustered genes in these species were presented by a bar-chart (Figure S5). There were 2,480 single copy orthologs, 588 multi copy orthologs, and 4,508 other orthologs in *F. luteovirens*. (Table S8). Among the 39,635 identified gene families in 30 species, 2711 gene families were shared by 30 fungi species, and 6,528 gene families were detected in *F. luteovirens*. A total of 1013 single copy gene families that were conserved across the species were extracted and aligned by MUSCLE. Then, the phylogenetic tree was constructed by RAxML. It was obvious that the constructed phylogenetic tree separated into three clades. One clade was Agaricales, under light blue background, and the rest two clades were fungus in Boletales and the outgroup fungus, respectively. Among the Agaricales, other two species in Agaricaceae (*A. bisporus*, *M. fuliginosa*) were the closest relatives of *F. luteovirens*. Besides, species in Physalacriaceae (*Armillaria gallica*, *Armillaria ostoyae*) and in Tricholomataceae (*Lepista nuda*, *Tricholoma matsutake*) were clustered into one small clade, respectively. And they were far away from *F. luteovirens* in the phylogenetic tree. These suggested that *F. luteovirens* belongs to Agaricaceae (Figure 2). Moreover, r8s and MCMCtree program was used to estimate the divergence time of all species. The final time tree showed that the divergence between Agaricaceae and other fungal families happened ∼176 Myr ago (Figure S6). After the formation of Agaricaceae family, unlike *A. bisporus* and *M. fuliginosa*, *F. luteovirens* was not followed the primary evolution routine. The speciation of it was ∼170 Myr ago, which was dramatically earlier than the split between *A. bisporus*, and *M. fuliginosa* (72 Myr ago), indicating that the evolution of c*F. luteovirens* was more independent. That’s may not only lead to the formation of unique traits in it, but also contribute to an ambiguous evolutionary relationship of *F. luteovirens*.

### The species-specific genes analysis

**Figure 3:**
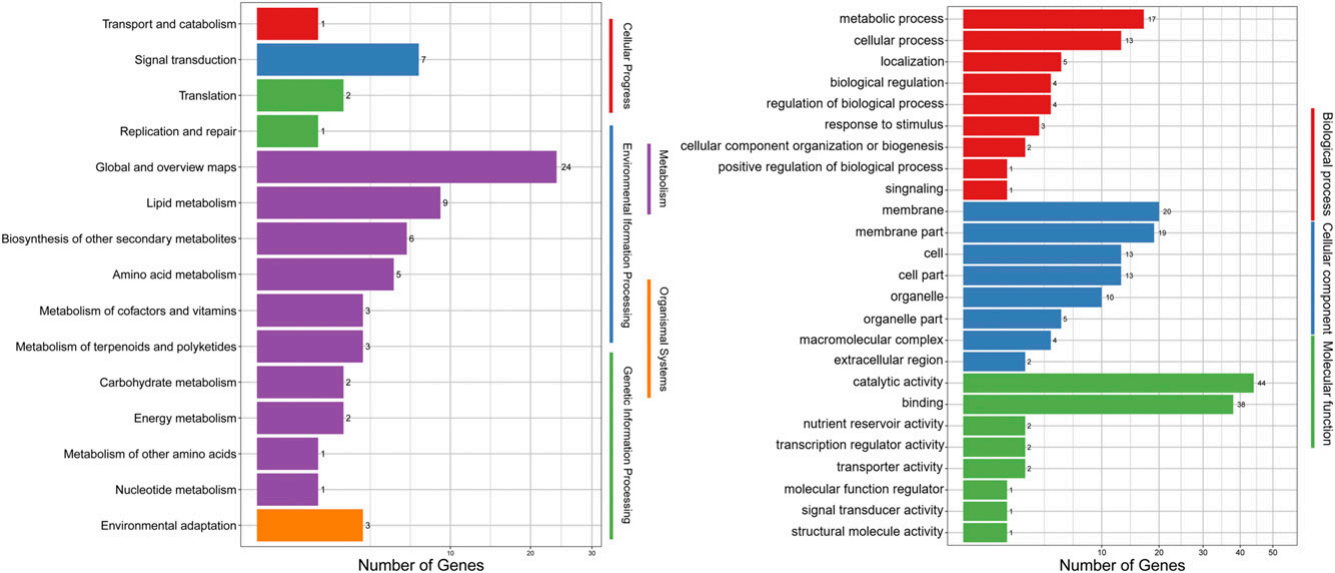
KEGG pathway classification and GO functional classification of species-specific genes in *Floccularia luteovirens* genome.

The gene families of *A. bisporus*, *F. luteovirens*, *T. matsutake*, and *M. fuliginosa* were selected to investigate the species-specific genes. *A. bisporus*, *M. fuliginosa* were relatively closed to *F. luteovirens* in the constructed phylogenetic tree, and *T. matsutake* was used as a control. In total, 4891 gene families were shared by all four fungus. Besides, 453, 357, 3,061, and 1,170 species-specific gene families belong to *A. bisporus*, *F. luteovirens*, *T. matsutake*, and *M. fuliginosa*, respectively (Figure S7). The 357 species-specific gene families containing 602 genes of *F. luteovirens* were picked for further GO and KEGG analysis. The KEGG pathway classification showed that they mainly classified in 5 categories. “Global and overview maps”, “Lipid metabolism”, and “Signal transduction” were the top 3 enriched KEGG pathways. Meanwhile, the GO functional classification implied that the predominant GO items in 3 major categories included “catalytic activity” “binding”, and “membrane” (Figure 3). These species-specific genes and gene families should contribute to the genetic and evolutionary characteristics of *F. luteovirens* on a large extent. Therefore, our annotation may help to excavate the major factors which could influence the speciation and differentiation of them.

## Discussion

In this study, we reported on a draft genome of *Floccularia luteovirens* which is the first sequenced genome of *Floccularia* genus. The assembled genome size was 28.8 Mb, comprising 183 contigs with a N50 contig size of 571 kb. A total of 8,333 gene models were predicted and 7,999 genes could be assigned to different protein databases. After the high-quality assembly and annotation, various of transposable elements and gene families were identified, which is conductive to analyze the genome expansion and evolution in the future. We also identified a number of CAZymes of *F. luteovirens*. This may help to reveal the key enzymes involved in the growth of its fruiting body and symbiosis, which has potential benefit for sustainable development of QTP alpine meadow ecosystem and artificial cultivation of the “Yellow mushroom”. According to our phylogenetic analysis, *F. luteovirens* belongs to Agaricaceae family, closing to *A. bisporus* and *M. fuliginosa*. The gene families and species-specific genes of *F. luteovirens* were sequentially extracted and annotated. Furthermore, our genome assembly strategy could be a practical model for analyzing other fungus genome. All the genomic information and resources will provide insights into further genetic investigation of *F. luteovirens*.
